# Influence of Culture Conditions on Bioactive Compounds in *Cordyceps militaris*: A Comprehensive Review

**DOI:** 10.3390/foods14193408

**Published:** 2025-10-01

**Authors:** Hye-Jin Park

**Affiliations:** Department of Veterinary Medicine, College of Veterinary Medicine, Konkuk University, Seoul 05029, Republic of Korea; nimpi79@konkuk.ac.kr

**Keywords:** *Cordyceps militaris*, fungal cultivation, bioactive compounds, solid fermentation, liquid fermentation, cultivation substrates

## Abstract

*Cordyceps militaris* (*C. militaris*) is a medicinal fungus renowned for its diverse therapeutic properties, largely attributed to bioactive compounds such as cordycepin, polysaccharides, adenosine, D-mannitol, carotenoids, and ergosterol. However, the production and composition of these metabolites are highly influenced by cultivation conditions, highlighting the need for systematic optimization strategies. This review synthesizes current findings on how nutritional factors—including carbon and nitrogen sources, their ratios, and trace elements—and environmental parameters such as oxygen availability, pH, temperature, and light regulate *C. militaris* metabolite biosynthesis. The impacts of solid-state fermentation (using grains, insects, and agro-industrial residues) and liquid state fermentation (submerged and surface cultures) are compared, with attention to their roles in mycelial growth, fruiting body formation, and secondary metabolite production. Special emphasis is placed on mixed grain–insect substrates and light regulation, which have emerged as promising methods to enhance cordycepin accumulation. Beyond summarizing advances, this review also identifies key knowledge gaps that must be addressed: (i) the incomplete understanding of metabolite regulatory networks, (ii) the absence of standardized cultivation protocols, and (iii) unresolved challenges in scale-up, including oxygen transfer, foam control, and downstream processing. We propose that future research should integrate multi-omics approaches with bioprocess engineering to overcome these limitations. Collectively, this review highlights both current progress and remaining challenges, providing a roadmap for advancing the sustainable, scalable, and application-driven production of bioactive compounds from *C. militaris*.

## 1. Introduction

**Figure 1:**
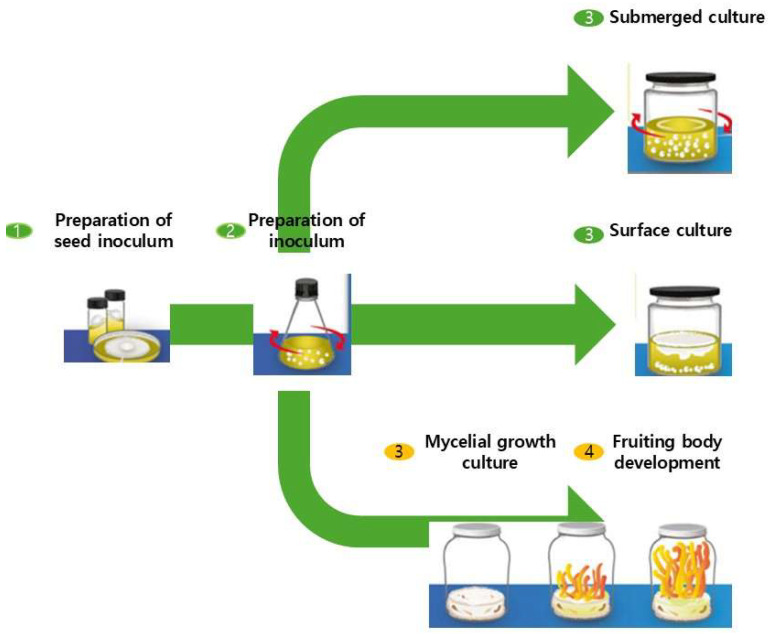
Solid and Liquid Fermentation of *Cordyceps militaris*. Color coding: Green after steps 3 and 4 represents liquid-state fermentation, whereas yellow after steps 3 and 4 indicates solid-state fermentation.

*Cordyceps militaris* (*C. militaris*) (CM; also called Yong Chong Cao) is widely used in traditional oriental medicine for its beneficial effects in treating conditions related to chronic fatigue syndrome [1], immune dysfunction [2,3], diabetes [4,5] inflammatory disorders, respiratory disorders [6,7,8], and cancer [9,10,11]. These effects are attributed to its various beneficial substances, including nucleotides (adenosine and cordycepin), polysaccharides, proteins, amino acids, peptides, ergosterol, and essential nutrients such as vitamins (B1, B2, B12, E, and K) and minerals (Ca, Fe, Mg, K, Se, Na, and Zn) [2,10]. Among these, cordycepin has been widely recognized for its anti-cancer and immunomodulatory effects, positioning it as a promising lead compound for pharmaceutical development; adenosine exhibits potent anti-inflammatory and neuromodulatory activity relevant for neurological and metabolic disorders; polysaccharides act as immunostimulants and prebiotics, making them highly suitable for functional food applications; D-mannitol contributes antioxidant and hepatoprotective effects; and ergosterol and carotenoids provide antioxidant and neuroprotective benefits [1,2,3,4,5,6,7,8,9,10,11]. The composition and concentration of these compounds within *C. militaris* can vary significantly depending on the culture medium used for artificial cultivation. Therefore, artificial cultivation of *C. militaris* has been developed by many researchers, as illustrated in Figure 1 [6,7].

Compared with other species such as *Cordyceps sinensis*, which faces host dependence and limited artificial cultivation, *C. militaris* demonstrates more stable fruiting body formation and higher metabolite yields under controlled culture conditions, making it an attractive alternative for research and commercial production [12,13]. However, despite these advantages, natural production is still constrained by low yields and high costs, creating a strong demand for optimized cultivation systems. Furthermore, clear strategies to bridge laboratory-scale findings with industrial production remain poorly defined, highlighting a pressing need for translational research. At the same time, market reports predict rapid growth of the functional mushroom sector, driven by consumer interest in immune-enhancing and anti-fatigue supplements.

The biosynthesis of these bioactive compounds is strongly influenced by nutritional and environmental parameters. Optimization of carbon/nitrogen ratio, pH, light, temperature, and oxygen supply not only supports fungal growth but also tailors metabolite profiles. For instance, blue light has been shown to upregulate genes associated with cordycepin and carotenoid synthesis, while nitrogen supplementation significantly modulates nucleoside production [14,15]. However, despite these opportunities for metabolic optimization, the natural production of *C. militaris* metabolites remains constrained by low yield and high production cost. This underscores the need for cultivation strategies that not only maximize biomass but also precisely regulate metabolic pathways to enrich functional compounds. To address these challenges, researchers have developed artificial cultivation systems employing both solid-state and liquid-state fermentation, as well as mixed substrates derived from cereals, legumes, and agro-industrial by-products (Figure 1). Such approaches aim to enhance yields and reduce costs to meet the growing global demand for nutraceuticals, functional foods, and pharmaceuticals derived from *C. militaris* [3]. Despite these advances, standardized cultivation protocols are still lacking or not publicly available, and the mechanistic regulation of metabolite biosynthesis—particularly at the transcriptional and metabolic flux levels—remains poorly understood. This gap has hindered the ability to establish clear strategies for translating laboratory-scale optimizations into reproducible, industry-scale processes. Furthermore, most findings are derived from small-scale experimental studies, and translation to commercial production continues to face unresolved bottlenecks, including oxygen transfer, foam control, and downstream processing.

Accordingly, the objective of this review is to integrate current insights into the nutritional and environmental regulation of *C. militaris* metabolite biosynthesis, to compare the relative strengths and limitations of solid-state and liquid-state fermentation strategies, and to evaluate substrate choices such as grains, insects, and agro-industrial residues. By explicitly linking mechanistic understanding with applied perspectives, this review aims to outline practical pathways for cost-effective, sustainable, and scalable production, while also identifying critical knowledge gaps and future research directions needed to advance both scientific insight and industrial application of *C. militaris*.

Color coding: Green represents liquid state fermentation, and yellow indicates solid-state fermentation.

## 2. Main Bioactive Compounds in *C. militaris* and Factors Affecting Their Production

Overall, the production of bioactive compounds in *C. militaris* is a dynamic process involving complex interactions between metabolic pathways and cultivation parameters. Optimizing these conditions—through strategic selection of carbon and nitrogen sources and careful control of C/N ratio, dissolved oxygen, pH, temperature, light, and fermentation time—enables targeted enhancement of specific metabolites for pharmaceutical and nutraceutical applications as summarized in Supplementary Table S1.

### 2.1. Cordycepin

Cordycepin (3′-deoxyadenosine) biosynthesis in *C. militaris* is strongly influenced by both nutritional and environmental factors. Among these, the type and concentration of carbon sources such as glucose, sucrose, maltose, and starch play a central role, as they directly affect the availability of adenosine precursors and consequently modulate cordycepin yields [16]. Nitrogen source is another key determinant: while organic nitrogen sources (e.g., yeast extract, peptone) generally provide efficient precursors for nucleotide synthesis, inorganic forms (e.g., NH_4_^+^, NO_3_^−^) vary in their effectiveness [17]. Importantly, the overall carbon-to-nitrogen (C/N) ratio dictates the metabolic balance—higher ratios tend to favor biomass accumulation, whereas moderately lower ratios shift metabolism toward secondary metabolite production, including cordycepin [16]. In addition to nutritional factors, environmental conditions critically shape cordycepin biosynthesis. Adequate dissolved oxygen (DO) levels are essential because cordycepin production is dependent on aerobic metabolism, and hypoxia markedly reduces yields. Similarly, maintaining pH and temperature within the optimal range is necessary to preserve enzymatic activity along the biosynthetic pathway. Trace metals also contribute: supplementation with Fe^2+^ or Zn^2+^ has been shown to significantly enhance production, with cordycepin titers reaching up to ~1.55 g/L under Zn treatment [18]. Light exposure further regulates biosynthesis, as blue light and dark conditions modulate the expression of enzymes associated with cordycepin pathways [19]. Beyond these physical and chemical parameters, metabolic precursors themselves can serve as stimulators. The addition of amino acids such as alanine, glycine, and aspartic acid has been reported to boost cordycepin accumulation [20,21]. Finally, the temporal dynamics of fungal growth are important: cordycepin concentration typically peaks during the stationary phase, following the exponential growth phase of mycelial proliferation [22]. Notably, higher cordycepin content per gram obtained from solid-state substrates may favor applications in nutraceuticals and functional foods, where fruiting body extracts are directly commercialized, whereas the higher volumetric yields achievable in liquid fermentation are more suitable for pharmaceutical-scale production that requires standardization and reproducibility. Together, these findings underscore the intricate interplay of nutrition, environment, and developmental stage in regulating cordycepin biosynthesis in *C. militaris*. This distinction provides practical guidance for selecting appropriate substrates and culture modes depending on the intended industrial application, thereby bridging experimental data with real-world production strategies.

Cordycepin (3′-deoxyadenosine) from *C. militaris* has been shown to exert an anti-tumor or anti-cancer action against human lung cancer cells, according to Park et al. [23]. Moreover, it inhibits liver cancer cell invasion and migration by downregulating CxCR4, which is a key factor in these processes [24]. Cordycepin alleviates fatigue induced by excessive exercise through its antioxidant effects, enhancing superoxide dismutase (SOD) activity and attenuating serum malondialdehyde (MDA) levels (*p*  <  0.01) [25]. Finally, it stimulates immune responses by enhancing cellular and humoral responses, which increases interleukin-4 (IL-4), interleukin-10 (IL-10), interleukin-12 (IL-12), T helper type 1 (Th1)/T helper type 2 (Th2) cytokines, and T cells (cluster of differentiation 4 (CD4)/cluster of differentiation 8 (CD8) while reducing interleukin-2 (IL-2) and transforming growth factor-beta (TGF-β) levels [7,26,27].

### 2.2. Polysaccharides

*Cordyceps* contain a substantial amount of polysaccharides, typically ranging from 3% to 8% of its total dry weight (DW) [28]. These polysaccharides are generally derived from the fruiting bodies, the mycelium produced via solid-state or submerged fermentation, and the fermentation broth [29]. The production of polysaccharides by *C. militaris* in fermentation media is influenced by multiple factors, including the types of carbon and nitrogen sources, the C/N ratio, the presence of inorganic salts and trace elements, and cultivation parameters, such as temperature, agitation speed, oxygen availability, light conditions, pH, and duration of fermentation [29]. *C. militaris*-derived exopolysaccharides (EPS) exhibit a wide range of biological activities, including immunomodulatory, antioxidant, antitumor, anti-inflammatory, antidiabetic, and prebiotic effects. Polysaccharides enhance the immune response by inducing macrophages to release NO, IL-1β, IFN-γ, TNF-α, and activating T/B lymphocytes, natural killer (NK) cells, and macrophage phagocytosis in vitro [30]. In addition, polysaccharides exhibit antitumor effects by inhibiting growth, inducing apoptosis, and arresting the tumor cell cycle at the G0/G1 and G2/M phases [30]. EPS also suppress inflammation by inhibiting key signaling pathways, improve insulin sensitivity, and support gut health by promoting beneficial microbiota. These multifunctional properties suggest that EPS have strong potential as a therapeutic agent [31].

### 2.3. Adenosine

Adenosine, another purine-derived nucleoside, shares early biosynthetic steps with cordycepin. Adenosine is recognized for its anti-inflammatory and immunomodulatory properties and exerts these effects via four receptor subtypes: A_1_, A_2_A, A_2_B, and A_3_ [32,33,34]. Among them, A_2_A plays a particularly prominent role: it is abundantly expressed in immune cells such as T lymphocytes, NK cells, macrophages, and dendritic cells, and its activation suppresses pro-inflammatory cytokine secretion while promoting anti-inflammatory responses [33,35]. The A_1_ receptor, although more widely distributed in neurons, cardiomyocytes, and kidney cells, also contributes to the inhibition of neutrophil activity [36]. Conversely, A_2_B is expressed on neutrophils, basophils, and macrophages, and can elicit either pro- or anti-inflammatory effects depending on the cellular context [37]. The A_3_ receptor is notably present on mast cells, neutrophils, and lymphocytes and is involved in suppressing degranulation and inflammatory responses [38]. Taken together, these data suggest that adenosine derived from *C. militaris* may exert its anti-inflammatory activity primarily through A_2_A receptor-mediated modulation of immune cell functions.

Several cultivation factors have been reported to enhance adenosine accumulation in *C. militaris*. Red light irradiation stimulates adenosine production more effectively than blue or white light [39], while supplementation with trace elements, such as Se, further increases yield in liquid culture [40]. Acidic culture conditions, particularly around pH 4.5–5.0, favor adenosine biosynthesis [41], and organic nitrogen sources such as yeast extract play a critical role, often serving as the most influential factor in submerged fermentation media [41]. The choice of cultivation substrate also significantly affects adenosine levels in fruiting bodies [42], which tend to increase as the organism matures, indicating that later harvest stages are more favorable [41]. Optimized strains and media formulations have achieved substantial improvements in adenosine content, and careful control of fermentation time is critical, as peak accumulation occurs during specific growth phases [41]. Finally, postharvest handling, particularly gentle drying methods, helps preserve adenosine levels, making both cultivation and processing strategies essential for maximizing its accumulation [43].

### 2.4. D-Mannitol

D-mannitol, commonly referred to as cordycepic acid, is one of the major bioactive metabolites produced by *C. militaris*. Chemically classified as a polyhydric alcohol (C_6_H_14_O_6_), it possesses diverse biological activities that contribute to the medicinal potential of the fungus. It plays roles in osmoregulation, antioxidant defense, and energy storage [44,45]. The mannitol content in *C. militaris* varies widely according to the strain and cultivation substrate, ranging from ~20 to nearly 190 mg/g DW [46,47]. Notably, mannitol exhibits strong antioxidant properties by scavenging free radicals and protecting cellular components such as hyaluronic acid from oxidative damage [48]. In addition, it has been shown to exert anti-inflammatory effects through the inhibition of lipid peroxidation and suppression of NF-κB signaling. Several studies have also reported its antitumor potential, particularly in combination with chemotherapeutic agents such as cisplatin, which enhances treatment efficacy and prolongs survival in animal models [49]. Furthermore, mannitol demonstrates hypolipidemic activity, comparable to that of standard lipid-lowering drugs, without adverse effects on liver function or HDL cholesterol levels [50]. It also plays a role in hepatoprotection, especially in conditions such as obstructive jaundice, and supports renal function by promoting prostaglandin-mediated vasodilation and diuresis [51]. These multifunctional properties highlight the pharmacological relevance of mannitol as a promising functional compound derived from *C. militaris*.

Mannitol production is sensitive to pH and C/N ratio; mildly acidic pH and balanced nutrient conditions tend to favor its accumulation. As mannitol biosynthesis branches from glycolytic intermediates, excessive carbon availability combined with limited nitrogen supply can channel flux toward mannitol rather than toward nucleotide-derived metabolites [14].

### 2.5. Carotenoids

Carotenoids, including cordyxanthins, are isoprenoid-derived pigments responsible for the characteristic orange coloration of fruiting bodies and cultures. Lan et al. demonstrated that a carotenoid-rich extract from *C. militaris* supported human retinal ARPE 19 cells under oxidative stress. The extract exhibited strong antioxidant and anti-apoptotic activities, effectively protecting these cells from H_2_O_2_-induced damage [52]. Research indicates that carotenoids, such as lutein and beta-carotene—even when sourced from *C. militaris*—can exert anti-aging effects by reducing oxidative stress and modulating pathways related to insulin-like growth factor (IGF-1), ROS production, and histone acetylation [44].

Their synthesis is stimulated by light exposure, especially in the blue to near-UV range, and is affected by pH and temperature. These pigments possess both antioxidant and potential neuroprotective properties. Light quality significantly affects carotenoid biosynthesis in *C. militaris*. Pink light (one-third blue, 450–460 nm; two-thirds red, 620–630 nm) has been shown to simultaneously maximize adenosine, cordycepin, and carotenoid accumulation. Likewise, blue light (17.3–64.3 lux) during fruiting body cultivation has been shown to markedly increase carotenoid content, reaching ~4.41 mg/g DW on oat-based medium [39]. These results suggest that both mixed-wavelength and blue light treatments can enhance carotenoid production, likely via the activation of light-responsive biosynthetic genes [39,53]. The blue-light photoreceptor gene CmWC-1 has been reported to regulate both carotenoid and cordycepin biosynthesis in *C. militaris*, with deletion mutants exhibiting reduced production of both compounds [54]. In various commercially obtained fruiting bodies of *C. militaris*, carotenoid content ranged from approximately 2.12 mg/mL to 3.85 mg/mL DW, with some lyophilized samples reaching as high as ≈5.01 mg/mL [55].

### 2.6. Ergosterol

Ergosterol is the major sterol component of fungal cell membranes. In *C. militaris*, it has been increasingly recognized as a bioactive metabolite with diverse pharmacological activities, including antioxidant and anti-inflammatory effects. By suppressing mediators such as NF-κB, iNOS, and COX-2, it alleviates inflammatory responses in macrophages [56], and it also functions as a free radical scavenger to protect cells from oxidative stress-induced injury [57]. In addition to these activities, ergosterol demonstrates immunomodulatory potential, as fungal sterols can regulate both innate and adaptive immune responses to support host defense [2]. Although ergosterol is a ubiquitous fungal sterol, its content in *C. militaris* has been employed as a useful biomarker for fungal growth and metabolic status. Moreover, previous studies have demonstrated its potential pharmacological activities—such as anti-inflammatory, antioxidant, and anti-proliferative effects—when extracted from *C. militaris* fruiting bodies or mycelia [58,59]. Ergosterol, the principal sterol in fungal membranes, is synthesized via the mevalonate pathway, and its production is influenced by oxygen availability—as several steps are oxygen-dependent—as well as by the composition of carbon and nitrogen sources. Moreover, it not only serves structural roles but also acts as a precursor for vitamin D_2_ upon UV exposure [8].

### 2.7. Bioactive Proteins

Among the bioactive proteins identified in *C. militaris*, cordymin—a ~10.9 kDa antifungal peptide—has demonstrated notable antioxidant and immunomodulatory effects [60]. CMP (*C. militaris* protein) induces apoptosis in murine hepatoma cell line BNL 1MEA.7R.1 [61], and lectin-like proteins are known for their hemagglutination and mitogenic activities [62]. Additionally, extracellular proteases and chitinases play key roles in nutrient acquisition and pathogenicity, particularly under insect-mimicking culture environments [63]. Protein biosynthesis in *C. militaris* is highly influenced by culture parameters. Organic nitrogen sources such as peptone and yeast extract significantly enhance total protein yield and extracellular enzyme production [44]. Exposure to blue light has been reported to upregulate chitinase, laccase, and oxidoreductase expression, which are associated with fungal defense and metabolite secretion [54], whereas temperature stress—both heat and cold—induces specific shock proteins that modulate the translational machinery [29]. Substrate composition also plays a critical role: grain-based substrates like rice, barley, and wheat not only supply carbohydrates but also provide essential amino acids that support protein biosynthesis. Liang et al. (2014) reported that these media, especially when supplemented with nitrogen sources, increased total protein content alongside co-metabolites such as mannitol and cordycepin [47]. In contrast, insect-based substrates such as silkworm pupae and beetle larvae stimulate the production of stress-responsive and hydrolytic proteins—including serine proteases, superoxide dismutase (SOD), thioredoxin, and lipid-metabolizing enzymes—which contribute to antioxidant defense, immune function, and membrane adaptation [58]. Moreover, solid-state fermentation utilizing insect–grain mixtures further upregulated stress-related proteins and transporters, indicating an adaptive response to the complex nutrient and oxidative environments created by such substrates [59,60].

### 2.8. Cross-Compound Integration and Industrial Implications

The biosynthesis of bioactive compounds in *C. militaris* is governed by an intricate interplay of nutritional, environmental, and developmental factors, with different metabolites often responding to overlapping signals. For instance, the carbon-to-nitrogen (C/N) ratio not only regulates cordycepin yields but also influences polysaccharide branching and mannitol flux, while light quality simultaneously affects carotenoid pigmentation, cordycepin pathways, and protein secretion through light-responsive genes (e.g., *CmWC-1*) [54]. Nitrogen source choice further demonstrates cross-compound effects: organic nitrogen sources (e.g., peptone, yeast extract) consistently enhance cordycepin, proteins, and extracellular enzymes, whereas inorganic salts such as ammonium sulfate can redirect flux toward mannitol production [14]. Dissolved oxygen availability is essential for cordycepin and adenosine biosynthesis, while hypoxia tends to favor polysaccharide accumulation [29]. These overlaps highlight that culture conditions should not be considered in isolation but as integrated determinants that shape the overall bioactive profile.

The biological activities of these compounds are diverse yet complementary. Cordycepin and adenosine modulate purinergic signaling, exerting anti-inflammatory, anticancer, and immunomodulatory effects [24,32]. Polysaccharides function as immunostimulants and prebiotics, mannitol contributes antioxidant, hepatoprotective, and hypolipidemic properties [30], while carotenoids and ergosterol provide antioxidant and neuroprotective activities. Bioactive proteins—including cordymin and CMP—add antifungal, anticancer, and immunomodulatory effects [60]. Collectively, this repertoire demonstrates the pharmacological versatility of *C. militaris* and the need for cultivation strategies that enhance multiple metabolites in parallel.

From an industrial perspective, the choice of fermentation mode and substrate should be tailored to the intended product profile. Solid-state fermentation (SSF) generally yields higher concentrations of cordycepin, polysaccharides, and carotenoids per gram of fruiting body, favoring nutraceutical and functional food applications where fruiting body powders and extracts are marketed [28,64,65,66]. In contrast, submerged fermentation (SmF) provides superior process control, reproducibility, and scalability, enabling consistent volumetric yields required for pharmaceutical applications [41,67,68]. Substrate design adds another layer of practicality: grain-based media are cost-effective and widely available, insect-based substrates—though more costly—offer unique metabolite diversity and protein expression, and agro-industrial by-products such as corn cob particles provide sustainable and competitive alternatives [69,70].

Taken together, these insights underscore the importance of a holistic optimization strategy that integrates environmental cues, substrate selection, and strain-specific responses. Such an approach can maximize yield, quality, and diversity of bioactive metabolites, while also aligning production with sustainability goals and bridging experimental findings with real-world applications in nutraceuticals, functional foods, and pharmaceuticals.

## 3. Solid-State Culture with Grain-Based and Insect-Based Substrates for Enhanced Growth and Bioactive Compound Production

### 3.1. Solid-State Fermentation and Substrates Roles in C. militaris Cultivation

#### 3.1.1. Grain-Based Culture Media for *C. militaris*

Substrate selection plays a crucial role in enhancing the growth and bioactive compound yield of *Cordyceps militaris*. Solid-state fermentation (SSF) using cereal grains—such as brown rice, wheat, oats, and barley—has been widely adopted because of their high nutritional value (amino acids, B vitamins, and minerals), low cost, and accessibility [44,71]. When supplemented with nitrogen sources such as yeast extract or peptone, these substrates can accelerate mycelial colonization, shorten the time to primordial initiation, and enhance fruiting body yield [47], while also promoting the biosynthesis of secondary metabolites including cordycepin, adenosine, D-mannitol, and polysaccharides. Among them, rice has been the most commonly used substrate due to its favorable nutrient profile and ease of handling in SSF [44].

Rice media supported stable mycelial growth and produced high yields of cordycepin (8.92 mg/g) and polysaccharides (34.5 mg/g), as reported by Lin et al. (2017) [72]. More recently, alternative substrates such as corncob particles (CCP), an agricultural by-product, showed even greater potential, with cordycepin and adenosine levels reaching 9.45 mg/g and 5.86 mg/g, respectively [72].

Recent developments in substrate optimization for *C. militaris* cultivation have emphasized the potential of cereal-based substrate combinations to enhance the production of bioactive compounds, particularly cordycepin [73]. Recent findings have suggested that cereal-based substrate combinations can significantly influence cordycepin biosynthesis and biomass accumulation in *C. militaris*. Multi-cereal formulations—particularly those incorporating rice, wheat (*Triticum* spp.), jowar (*Sorghum bicolor*), bajra, ragi (*Eleusine coracana*), and sugarcane bagasse—have been systematically evaluated for their effects on metabolite yield. Among the tested combinations, mixtures such as rice–wheat–jowar (6.6 g each), rice–wheat–jowar–sugarcane bagasse (5 g each), rice–wheat–jowar–ragi–bajra (4 g each), and rice–wheat–jowar–bajra–sugarcane bagasse (4 g each) yielded significantly higher cordycepin and polysaccharide concentrations compared to the rice-only control (*p* < 0.05) [73]. Such combinations also produced marked increases in fresh biomass, underscoring the value of blending grains to optimize metabolite yields. In addition, agricultural by-products such as cottonseed shells, sawdust, and spent mushroom substrates have been successfully evaluated as low-cost alternatives [69].

Although adenosine levels showed only marginal increases across these combinations, the correlation between biomass enhancement and cordycepin production underscores the value of blending substrates to optimize metabolite yields [73]. Collectively, these results highlight the potential of grain-based media as sustainable and cost-effective options for large-scale SSF of *C. militaris*, particularly for functional food and pharmaceutical applications.

In efforts to enhance the productivity of *C. militaris*, various agricultural waste-derived solid substrates have been evaluated for their efficacy in supporting fruiting body formation and biosynthesis of bioactive compounds. A comparative study investigated the effects of substrates, such as cottonseed shells (CS), corncob particles (CCP), Italian poplar sawdust (IPS), and spent substrates (SS) from *Flammulina velutipes*, with rice serving as a conventional control. These solid-state media were formulated by blending agricultural residues (e.g., CS or CCP) with wheat bran and rice bran, followed by moistening with a defined nutrient solution and subsequent sterilization. Each cultivation unit received 40 g of dry substrate, whereas the rice control group received 20 g per unit [72].

Beyond rice, alternative substrates such as chickpeas have also shown promising results [74]. Xiao et al. reported that *C. militaris*-fermented chickpeas exhibited significantly increased levels of crude protein, true protein, and essential amino acids, highlighting their potential as a novel functional food [66]. Compared with wild-harvested *Cordyceps*, solid-state fermentation (SSF) of *C. militaris* provides significant advantages for industrial application. Wild materials often suffer from inconsistent metabolite levels due to host insect variability, seasonal factors, and geographic influences, making standardization and large-scale production difficult. In contrast, SSF offers controllable and reproducible cultivation conditions, enabling higher and more stable yields of key bioactive compounds such as cordycepin, adenosine, and polysaccharides. This approach minimizes contamination risks, reduces heavy-metal and microbial burdens, and allows compliance with Good Manufacturing Practice (GMP) and Hazard Analysis and Critical Control Point (HACCP) standards. Such standardization and safety are particularly critical for pharmaceutical use, where purity, reproducibility, and regulatory compliance are essential. At the same time, SSF can be performed on edible substrates like rice, millet, or germinated soy, which supports the development of whole-biomass products suitable for functional food applications. Thus, while SSF facilitates the production of purified bioactives for pharmaceutical formulations, it also provides a practical route to generate safe, scalable, and value-added products for the functional food market (Table 1) [65,66,69,75,76].

#### 3.1.2. Insect-Based Culture Media for *C. militaris*

Insect-based substrates provide a nutrient-rich and physiologically relevant environment for *C. militaris*, more closely resembling natural hosts than cereal media. Experimental work has demonstrated that environmental conditions can strongly influence infection dynamics. For example, larvae injected with *C. militaris* blastospores and maintained at 25 °C showed successful metamorphosis with low mortality, comparable to controls, whereas pupae kept at 15 °C exhibited markedly higher mortality and visible stromata formation in all mummified pupae [29]. These findings suggest that low-temperature exposure can activate latent infections, while a subset of insects (9–14%) displayed mycelial overgrowth after adult emergence regardless of temperature [29]. Such results highlight that both environmental and host-related factors play critical roles in fungal development.

Adenosine and cordycepin production by *C. militaris* varied significantly depending on the insect substrate used for cultivation. Efforts to enhance *C. militaris* production have focused on boosting cordycepin levels due to its broad biological activity. While grain substrates are common for economic reasons, insect-based cultivation yields higher quality products because insects resemble natural hosts and contain more protein and fat [64,78,80]. Tang et al. confirmed that both the total fatty acid content and specific composition, especially oleic acid, strongly influence cordycepin biosynthesis by upregulating cns1 and cns2 transcription [70,81]. Previous research has shown that fatty acids and oils promote fungal metabolite synthesis and influence cell membrane permeability, potentially facilitating cordycepin efflux and sustained production [81,82]. Accordingly, insects with higher oleic acid content are considered superior substrates for cordycepin-rich cultivation.

Substrate-dependent variation is well documented. For instance, cultivation on *Brihaspa atrostigmella* (*B. atrostigmella*)—showed the highest levels of both adenosine (1.062 mg/g) and cordycepin (2.932 mg/g), suggesting enhanced metabolic activity when grown on this specific host. In contrast, the lowest adenosine levels were observed in samples derived from the solid residues of *C. militaris* cultivated on *Bombyx mori* and *B. atrostigmella*, indicating that certain residue forms may limit nucleoside biosynthesis [79]. A similar trend was observed for cordycepin content, which ranged from 0.207 mg/g in solid based residues of *C.militaris* cultivated on *Oxya chinensis (Grasshoppers)* (SOC) to 2.932 mg/g in the fruiting bodies of *C. militaris* cultivated on *B. atrostigmella* (FBA). These results highlight the critical role of substrate composition and structure in modulating secondary metabolite accumulation [79]. Statistical analyses also indicated consistent metabolic output within host groups, underscoring the importance of host-specific nutritional and biochemical factors in shaping secondary metabolite accumulation.

Hosts from the order *Lepidoptera*, including the ghost moth, are characterized by high lipid and protein contents, which favor robust fungal growth and enhance cordycepin biosynthesis, a compound noted for its anti-cancer and immune-regulatory effects [58]. Similarly, *Coleoptera*, such as beetles, provide a chitinous exoskeleton that supports the production of chitinase, an enzyme crucial for fungal structural integrity and immune-enhancing effects [83]. *Hymenoptera*, including bees and wasps, possess unique fatty acid and sterol profiles that augment the pharmacological properties of *C. militaris* preparations [84]. Among edible insects, *Tenebrio molitor*, *Allomyrina dichotoma,* and silkworm pupae, showed significantly enhanced production of bioactive compounds. Owing to their abundant nutrients, silkworm pupae are widely used as hosts to enhance the synthesis of cordycepin and polysaccharides that exhibit notable anti-inflammatory and immune-enhancing activities [85]. It has been reported that *C. militaris* grown on *A. dichotoma* larvae accumulated cordycepin up to 89.5 mg/g—34 times higher than silkworm pupae [70]. From an industrial perspective, insect-based cultivation provides superior yields of pharmacologically active metabolites, making these substrates attractive for pharmaceutical development where potency and metabolite richness are prioritized. However, scalability is limited by cost, availability, and consumer acceptance. Importantly, responses to insect substrates are strain-dependent: not all *C. militaris* isolates produce comparable yields, and genetic background strongly influences metabolite accumulation (Table 1). This limitation must be acknowledged as a key barrier to industrial scaling and highlights the necessity of precise strain–substrate matching strategies.

#### 3.1.3. Mixed Grain- and Insect-Based Culture Media for *C. militaris*

*C. militaris*, valued for its diverse pharmacological activities, can be cultivated on composite substrates that integrate the nutritional advantages of both grains and insects. In Korea, a large-scale study evaluated 113 wild isolates first on Sabouraud dextrose agar with yeast extract (SDAY), and subsequently on a sterilized mix of brown rice and silkworm pupae. While strain-dependent differences were observed, several isolates exhibited robust mycelial growth, though stromata formation was limited under these conditions [86]. In a mixed grain–insect medium composed of various rice types (sticky, black, brown, and polished) supplemented with silkworm pupal powder, *C. militaris* showed good mycelial growth, with strain 17944 performing best; however, no stromata were produced under these conditions. Interestingly, *P. tenuipes* (an entomopathogenic fungus belonging to the family *Clavicipitaceae*) formed stromata only on sticky rice enriched with pupal powder or on whole silkworm pupae, further underscoring the importance of substrate composition in determining developmental outcomes (Table 1) [87].

More recently, Sibounnavong et al. (2024) reported that combining *Gryllus bimaculatus* (cricket) or other insect meals with brown rice not only enhanced adenosine and cordycepin yields but also increased α-glucosidase and α-amylase inhibitory activities, highlighting their promise for functional food applications [88]. Such findings suggest that insect-enriched media stimulate metabolic pathways associated with nucleoside and enzyme production more effectively than cereal substrates alone, thereby broadening the pharmacological spectrum of *C. militaris*.

From an industrial perspective, mixed media offer dual advantages: grains such as rice provide cost-effectiveness and scalability, while insect-derived components contribute unique amino acid and lipid profiles that broaden the metabolite spectrum. However, the relatively higher cost and limited availability of insect substrates as well as variability in fungal response across strains remain practical challenges for large-scale application. Not all *C. militaris* isolates perform optimally on mixed media, and strain-dependent differences represent a key limitation for scaling and industrial standardization. Taken together, integrating grains with nutrient-dense insect materials represents a sustainable strategy to improve both yield and biochemical diversity of *C. militaris*. When appropriately matched to specific fungal strains, such mixed-substrate approaches can bridge the gap between the scalability of grain-based systems and the potency of insect-based cultivation, thereby offering a versatile platform for both functional food and pharmaceutical applications [89].

### 3.2. Solid-State Fermentation and the Role of Physical Conditions

#### 3.2.1. Solid-State Fermentation and the Role of Temperature in *C. militaris* Cultivation

Most *C. militaris* cultures showed a temperature optimum of 20 °C, and only one of the Chinese isolates had an optimum of 25 °C. The mycelial growth of all cultures was impeded at 27.5 °C and completely ceased at 30 °C. Meanwhile, all of the isolates assayed exhibited active growth under cold conditions (5–15 °C) [29].

The development and production of cordycepin using *C. militaris* have been widely investigated through solid-state fermentation (SSF) on various substrates, including rice, wheat, and oat, with the optimal production temperature identified as 25 °C, suggesting that cordycepin is a non-growth-associated metabolite [75,90]. Previous studies have also reported cordycepin yields ranging from 93 to 544 mg/L at 25 °C in various *Cordyceps* strains, which is consistent with the findings of the present study [91].

#### 3.2.2. Solid-State Fermentation and the Role of Light in *C. militaris* Cultivation

SSF is a widely used technique for cultivating *C. militaris*, employing solid substrates such as brown rice, maize, soybeans, rye, various cereal grains, or insect-based biomass. SSF is typically conducted in small-scale containers, such as glass jars or trays, and comprises three distinct stages: (i) mycelial colonization, (ii) fruiting body development, and (iii) cordycepin accumulation. Each phase requires precise environmental control, including temperature, humidity, and light exposure. During the initial mycelial stage, growth is carried out in dark conditions at 19–21 °C for approximately one to two weeks. Fruiting body induction requires the introduction of light–dark cycles to simulate natural day and night rhythms [92]. Light exposure of 500–1000 lux for 8–12 h per day at a temperature range of 16–23 °C and relative humidity of 70–95% has been shown to facilitate robust fruiting body formation [93].

Light plays a crucial regulatory role in the final phase of cordycepin biosynthesis. Studies have demonstrated that specific light wavelengths significantly influence secondary metabolite production. For instance, Chiang et al. reported that green LED light (526–531 nm) enhanced cordycepin synthesis to 2.89 mg/g in *C. militaris* grown on brown rice. Moreover, a 12-h light period using fluorescent light yielded cordycepin levels as high as 3.97 mg/g. Notably, a specific LED spectrum combining red and blue wavelengths in a 3R:3B ratio led to the highest reported cordycepin content (30 mg/g). These findings underscore the significance of substrate composition and photoperiod regulation for maximizing the biosynthetic capacity of *C. militaris* under SSF conditions [94].

### 3.3. Solid-State Fermentation and the Role of Minerals in the Growth of C. militaris Mycelia and Fruiting Bodies

Comparative analysis of different inorganic salts in liquid culture (23 °C, 7 days) indicated that K_2_HPO_4_ and MgSO_4_·7H_2_O were particularly favorable for *C. militaris* mycelial growth and biomass accumulation, consistently ranking among the most effective supplements under the tested conditions [63]. Proteomic and metabolic pathway analyses have suggested that Mg plays a key role in fruiting body formation and in enhancing adenosine (a precursor) production, whereas sulfate ions (SO_4_^2−^) are critical for stimulating cordycepin biosynthesis. Consistently, supplementation with MgCl_2_ promoted fruiting body development and adenosine accumulation but had little effect on cordycepin levels, while sulfate-rich formulations markedly increased cordycepin production. In contrast, high concentrations of chloride ions (Cl^−^) have been reported to potentially inhibit cordycepin biosynthesis [95]. Compared with ultrapure water, deep ocean water (DOW) has been shown to significantly increase *C. militaris* biomass and adenosine production in submerged culture (*p* < 0.05), and in solid-state culture, cordycepin production was shown to have a positive dose–response to DOW concentration. Ion-resolved analyses indicate that Mg^2+^, Na^+^, Ca^2+^, Fe^2+^, and NO_3_^−^ promote cordycepin (with Cl^−^ inhibitory), while Mg^2+^, Na^+^, K^+^, Ca^2+^, Fe^2+^, and SO_4_^2−^ promote adenosine. A synthetic chloride-salt mixture (MgCl_2_, NaCl, KCl, CaCl_2_, FeCl_2_) can reproduce DOW-level cordycepin yields, implying that ionic composition and balance—rather than the water matrix—govern metabolite biosynthesis; thus, optimizing Mg^2+^/Fe^2+^/NO_3_^−^ while minimizing Cl^−^ is a practical lever to enhance growth and nucleoside production [96]. High copper (Cu) concentrations in certain DOW formulations have been suggested to inhibit cordycepin production by suppressing the cAMP signaling pathway, likely through interference with GTP binding and adenylate cyclase activity. Similarly, strontium (Sr), analogous to Ca, has been implicated in the regulation of adenylate cyclase activity, thereby linking it to the modulation of the cAMP pathway and cordycepin biosynthesis [95]. Low concentrations of fluoride, particularly 0.01 mM potassium fluoride, were shown to promote fruiting body growth and enhance bioactive compound accumulation in *C. militaris*, leading to increased carotenoid content, antioxidant activity, and improved anticancer effects in human osteosarcoma cells [97]. Wen’s group optimized the solid-state fermentation (SSF) of *C. militaris* on brown rice using both one-factor-at-a-time and orthogonal experimental designs. Their results demonstrated that medium composition strongly determines the balance between fruiting body yield and cordycepin accumulation. A glucose-rich formulation with moderate levels of peptone and mineral salts supported robust fruiting body development, whereas a more nitrogen-balanced formulation redirected metabolism toward cordycepin biosynthesis. These findings underscore an important practical implication: nutrient formulation can be strategically adjusted depending on whether the production goal is bulk biomass (fruiting bodies) for functional foods or enhanced nucleoside yields for nutraceutical or pharmaceutical applications. By establishing reproducible medium conditions, this study provides a foundation for scaling SSF systems beyond laboratory trials, where the dual goals of maximizing yield and ensuring economic feasibility must be balanced for industrial adoption [65]. Nitrate (NO_3_^−^) has been reported to play a direct role in promoting cordycepin production [98]. Se-enriched cultivation of *C. militaris* via sodium selenite (Na_2_SeO_3_) supplementation modulated fruiting-body chemistry: SOD activity and the contents of cordycepin, cordycepic acid (D-mannitol), and organic Se increased in a concentration-dependent manner, whereas adenosine and *Cordyceps* polysaccharides were enhanced but not strictly proportional to dose. On wheat substrate with 18.0 ppm sodium selenite, increases versus non-Se controls and wild *C. sinensis* were, respectively: SOD +121%/+145%, cordycepin +124%/+74%, cordycepic acid +325%/+520%, adenosine +130%/+284%, polysaccharides +121%/+145%, and total amino acids +157%/+554%. Fruiting bodies accumulated organic Se to 6.49 mg/100 g, indicating sodium selenite fortification is an effective lever to co-enhance antioxidant capacity and key bioactive metabolites in *C. militaris* (Table 2) [99].

## 4. Liquid State Fermentation

### 4.1. Overview of Liquid State Fermentation

Liquid state fermentation, also known as submerged fermentation, is a scalable and efficient alternative to the traditional solid-state methods for cultivating *C. militaris*. In addition, it is widely employed as a pre-culture stage prior to the solid-state cultivation of *C. militaris* and other filamentous fungi. Liquid mycelial suspensions enable precise inoculum control, leading to consistent results, faster colonization, and reduced contamination risks [76,100]. In this system, fungal mycelia are grown in a liquid nutrient medium under controlled conditions, enabling faster growth and higher yields of bioactive compounds owing to uniform nutrient distribution and improved oxygenation. In the referenced study, the glucose–peptone–beef extract–yeast extract (GPBY) liquid medium, consisting of 20 g L^−1^ glucose, 5 g L^−1^ peptone, 3 g L^−1^ beef extract, and 1 g L^−1^ yeast extract, was specifically used for liquid spawn preparation and not for fruiting body production. This design enabled the direct evaluation of how solid substrates influence fungal development and secondary metabolite synthesis, providing valuable insights for sustainable and cost-effective cultivation using agro-industrial byproducts [72]. Culturing *Cordyceps* in liquid media offers improved control over nutrient availability and environmental conditions, often leading to higher concentrations of bioactive compounds compared to cultivation on solid media [10].

### 4.2. Nutrient Composition of Liquid Fermentation Media

The formulation of the liquid fermentation medium is a critical factor that influences biomass yield and metabolite production. Commonly used carbon sources include glucose, sucrose, and maltose, which serve as primary energy substrates. Nitrogen is typically supplied through organic compounds such as yeast extract, peptone, or soybean hydrolysate. Inorganic salts like potassium dihydrogen phosphate (KH_2_PO_4_) and magnesium sulfate (MgSO_4_·7H_2_O) are also added to support cellular metabolism and enzymatic activity [17]. The initial pH of the medium is usually adjusted to 6.5–7.0, which has been shown to be favorable for both growth and secondary metabolite biosynthesis [75]. Mechanistic studies have shown that iron supplementation (Fe^2+^) can redirect purine metabolism toward the AMP/adenosine branch, thereby enhancing cordycepin biosynthesis. This effect is linked to the upregulation of adenylosuccinate synthetase (purA) and corresponding shifts in purine metabolic flux [18]. Supplementation with metabolic precursors (e.g., adenosine) or elicitors (e.g., rotenone, L-alanine) has been reported to further enhance biosynthesis, while the addition of specific mineral salts (e.g., K, Mg) or DOW–derived elements can also stimulate cordycepin production [80]. The carbon-to-nitrogen (C/N) ratio is another critical driver of metabolic balance. Moderate ratios favor both cordycepin and adenosine accumulation, while excessively low values disrupt productivity [18]. Yeast extract is consistently reported as the most effective organic nitrogen source for extracellular polysaccharides (EPS) and cordycepin, whereas corn steep powder particularly favors adenosine production [101]. Plant oils, while not directly enhancing nucleoside biosynthesis, improve mycelial growth and EPS formation, indirectly contributing to overall productivity [102]. Collectively, these findings suggest that the liquid fermentation medium should be designed with a balanced C/N ratio, appropriate organic nitrogen supplementation, and carefully selected mineral additives. By integrating metabolic precursors and elicitors with optimized baseline nutrients, researchers can shift metabolic flux toward higher yields of target metabolites. This approach underscores the importance of linking nutrient formulation to underlying regulatory mechanisms, rather than relying solely on empirical optimization.

### 4.3. Liquid Fermentation Conditions: Submerged vs. Surface Culture

Standard cultivation parameters for *C. militaris* in liquid media include an incubation temperature of 22–25 °C and agitation speeds of 120–150 rpm, which ensure adequate oxygen transfer and prevent mycelial sedimentation for optimal biomass accumulation. The cultivation period typically ranges from 5 to 14 days, depending on the strain, medium composition, and target compound [67,68]. Compared to solid-state fermentation (SSF), liquid fermentation offers several advantages, including shorter cultivation times, reduced space requirements, higher productivity, and simplified downstream extraction since bioactive compounds are secreted directly into the culture broth [44,103,104]. In this method, *C. militaris* grows exclusively as mycelia without forming fruiting bodies, thereby reducing the total cultivation time from approximately 60 to 15 days [44,105]. Furthermore, liquid culture does not require light/dark cycling or strict humidity control, making it a technically convenient and industrially favorable platform for the large-scale production of functional compounds derived from *C. militaris* (Table 3).

Recent studies have further classified liquid cultures into two types: submerged and surface (static) fermentations. In contrast, LSC supports growth at the air–liquid interface, forming dense mats under static conditions, where a biofilm forms on the liquid surface, with some mycelial biomass settling at the bottom. Although surface culture has been reported to yield higher levels of certain metabolites [106], it is associated with longer cultivation times and scalability challenges, limiting its industrial use [93]. LSC of *C. militaris* yielded 4.92 g/L cordycepin—vastly exceeding submerged culture (1 mg/L)—due to hypoxia-induced upregulation of purine metabolism and related genes. Transcriptomic analysis identified key enzymes and pathways that could be targeted to optimize cordycepin biosynthesis [106]. These hypoxic environments can stimulate certain metabolic pathways, leading to higher relative levels of selected metabolites. However, LSC requires longer cultivation periods and is less scalable, limiting its industrial adoption [103,106].

SmF, in contrast, maintains dispersed mycelia under agitation, ensuring oxygen transfer and nutrient dissolution [92]. This method provides precise process control, reproducibility, and compatibility with bioreactor systems, making it the preferred platform for large-scale production [10]. Submerged cultures are easily scalable using fermentation tanks and offer a shorter and more controllable fermentation process [93]. As a result, current industrial efforts are primarily focused on optimizing submerged liquid culture systems for efficient and standardized production of *C. militaris*-derived compounds [44].

Efficient process control is essential for maximizing cordycepin production in *C. militaris* submerged culture. Mechanistically, oxygen availability is a key driver: high dissolved oxygen (DO) favors nucleotide-derived metabolites such as cordycepin and adenosine, while oxygen limitation redirects metabolic flux toward polysaccharide biosynthesis [104,106]. Maintaining optimal pH and temperature is equally important; values near pH 6.0 and moderate temperatures (20–25 °C) have been reported to support balanced growth and metabolite accumulation in various *Cordyceps* species. Foam formation during aerated fermentation presents a dual risk of reducing oxygen transfer efficiency and increasing contamination potential; therefore, careful selection and dosing of antifoaming agents is recommended, ideally integrated into DO control strategies to maintain consistent oxygen delivery [17]. Optimal culture conditions for *C. militaris* vary depending on the target metabolites. Relatively low pH favored mycelial growth, EPS, and cordycepin production.

In addition, light quality and photoperiod can modulate cordycepin biosynthesis, even in liquid culture systems; blue light or mixed red–blue LED illumination (e.g., R:B = 3:7, 12 h/day) has been shown to enhance production [40]. Chiang et al. investigated the influence of different light-emitting diode (LED) conditions on mycelial biomass and cordycepin production and reported that a combination of blue and green light was most effective for cordycepin accumulation, whereas red light preferentially enhanced biomass formation [107].

A two-stage fermentation system that combined an initial aerobic shake-flask phase with a subsequent static culture phase was shown to markedly improve cordycepin production. Optimization studies (e.g., Box–Behnken design) identified suitable conditions of pH, nitrogen supplementation, and cultivation time, which synergistically enhanced yields. This strategy demonstrates how sequential adjustment of culture phases can redirect metabolic flux, thereby supporting higher cordycepin accumulation compared to conventional single-stage systems [41].

## 5. Conclusions

*C. militaris* has emerged as one of the most promising medicinal fungi due to its diverse repertoire of bioactive metabolites, including cordycepin, polysaccharides, adenosine, carotenoids, and ergosterol. Accumulating evidence demonstrates that both nutritional and environmental factors—such as substrate composition, light, temperature, pH, and oxygen availability—play critical roles in regulating metabolite biosynthesis. Optimizing these parameters through solid-state and liquid fermentation strategies has been shown to significantly enhance yields of both fruiting bodies and mycelia, thereby broadening the possibilities for scalable production (Supplementary Table S2 [20,22,41,44,65,100,106,108]).

Recent advances highlight the potential of mixed substrates and precision regulation of cultivation conditions to tailor the metabolic profile of *C. militaris*. These strategies pave the way for practical applications in nutraceuticals, functional foods, and pharmaceuticals.

Nevertheless, several limitations remain. The lack of standardized cultivation protocols and variations across production systems hinder reproducibility and large-scale consistency. Moreover, the functional correlation between metabolite diversity and biological activity is still not fully understood. Economic feasibility and cost-effective scaling also remain significant challenges. In particular, engineering constraints such as efficient oxygen transfer in bioreactors, foam management during aerated fermentation, and downstream processing for metabolite purification present critical bottlenecks that must be addressed before wide-scale industrial application can be realized.

Future research should therefore focus on integrating metabolomics, genomics, transcriptomics, and bioengineering approaches to uncover the regulatory networks of metabolite biosynthesis and to design targeted cultivation strategies. Addressing these limitations through interdisciplinary efforts will be essential for unlocking the full therapeutic and commercial potential of *C. militaris*.

## Figures and Tables

**Table 1:** Comparison of bioactive compound content in *Cordyceps militaris* cultivated on grain- and insect-based media.

Substrate	Cordycepin (mg/g)	Adenosine (mg/g)	D-Mannitol (mg/g)	Crude Polysaccharides (mg/g)	Cost-Effectiveness/Sustainability	Reference
rice (Control)	8.92	4.57	150	34.5	Moderate cost, widely available	[72]
corn cob particles (CCP)	9.45	5.86	100	26.9	Very cost-effective, agro-industrial by-product	[72]
cottonseed shells (CS)	8.6	3.98	120	23.4	Low-cost agricultural waste	[72]
Italian poplar sawdust (IPS)	2.7	1.22	80	<17.25 (less than half of control)	Low-cost residue	[72]
spent substrate (SS) by the mushroom *Flammulina velutipes*	2.6	1.49	75	<17.25	Low-cost residue	[72]
rice (*Oryza sativa*)	1.1	2	-	-	Moderate cost, widely available	[69]
rice, wheat (*Triticum*), and jowar (*Sorghum bicolor*) (1:1:1)	1.6	2.8	-	-	Moderate cost, widely available	[69]
rice, wheat, and bajra (*Pennisetum glaucum*)	1.1	2.1	-	-	Moderate cost, widely available	[69]
rice, wheat, jowar, and bajra	1.7	2.9	-	-	Moderate cost, widely available	[69]
rice, wheat, bajra, and ragi (*Eleusine coracana*)	1.4	3	-	-	Moderate cost, widely available	[69]
rice, wheat, bajra, and ragi (*Eleusine coracana*)	1.8	2.9	-	-	Moderate cost, widely available	[69]
rice, wheat, jowar, and sugarcane bagasse	2	3.3	-	-	Moderate cost, widely available	[69]
rice, wheat, jowar, ragi, and bajra	2.1	2.8	-	-	Moderate cost, widely available	[69]
rice, wheat, jowar, bajra, and sugarcane bagasse	2	2.5	-	-	Moderate cost, widely available	[69]
rice, wheat, jowar, bajra, sugarcane bagasse, and ragi	1.9	2.8	-	-	Moderate cost, widely available	[69]
brown rice	8.21	0.5	117.94	-	Moderate cost, widely available	[47]
brown rice + 1% peptone	5.83	0.54	167.97	-	Moderate cost, widely available	[47]
brown rice + 1% yeast extract	4.74	0.66	124.98	-	Moderate cost, widely available	[47]
brown rice + 1% ammonium sulfate	10.05	0.48	90.25	-	Moderate cost, widely available	[47]
brown rice + 1% monosodium glutamate	11.93	0.36	121.13	-	Moderate cost, widely available	[47]
plumule rice	10.1	0.42	125.85	-	Moderate cost, widely available	[47]
plumule rice + 1% peptone	11.45	0.42	123.23	-	Moderate cost, widely available	[47]
plumule rice + 1% yeast extract	6.84	0.68	152.35	-	Moderate cost, widely available	[47]
plumule rice + 1% ammonium sulfate	8	0.64	135.05	-	Moderate cost, widely available	[47]
plumule rice + 1% monosodium glutamate	16.44	0.24	109.86	-	Moderate cost, widely available	[47]
wheat	19.12	0.48	101.76	-	Moderate cost, widely available	[47]
wheat + 1% peptone	13.54	0.54	110.82	-	Moderate cost, widely available	[47]
wheat + 1% yeast extract	13.59	0.55	125.04	-	Moderate cost, widely available	[47]
wheat + 1% ammonium sulfate	13.05	0.52	92.62	-	Moderate cost, widely available	[47]
wheat + 1% monosodium glutamate	22.14	0.61	198.35	-	Moderate cost, widely available	[47]
pearl barley	3.96	0.43	103.32	-	Moderate cost, widely available	[47]
pearl barley + 1% peptone	8.96	0.48	112.25	-	Moderate cost, widely available	[47]
pearl barley + 1% yeast extract	2.09	0.44	96.56	-	Moderate cost, widely available	[47]
pearl barley + 1% ammonium sulfate	10.92	0.61	141.32	-	Moderate cost, widely available	[47]
pearl barley + 1% monosodium glutamate	14.5	0.4	134.71	-	Moderate cost, widely available	[47]
brown rice medium	6.63	-	-	-	Moderate cost,	[77]
soybean	8.33	-	-	-	Moderate cost	[74]
chickpea	11.12				Moderate cost	[74]
black bean	10.43				Moderate cost	[74]
silkworm pupae medium	8.1	-	-	-	High cost, limited scalability	[77]
*Bombyx mori*	0.2	-	-	-	High cost, limited scalability	[78]
*Protaetia brevitarsis*	4.3	-	-	-	High cost, limited scalability	[78]
*Tenebrio molitor*	0.3	-	-	-	High cost, limited scalability	[78]
*Allomyrina dichotoma*	8.9	-	-	-	Expensive, less scalable	[78]
*Gryllus bimaculatus*	1.5	-	-	-	Expensive, less scalable	[78]
*Locusta migratoria*	3.4	-	-	-	Expensive, less scalable	[78]
*Brihaspa atrostigmella*	2.932	1.062	-	-	Expensive, less scalable	[79]
*Allomyrina dichotoma* larva	89.5	-	-	-	Expensive, less scalable	[70]

“-“ denotes missing data. Substrate concentrations are expressed as grams per 100 mL or per flask, depending on the culture method.

**Table 2:** Reported effects of minerals on *Cordyceps militaris* bioactive compound production.

Mineral	Reported Effect	Notes	Reference
Zn^2+^	enhances cordycepin production (up to ~1.55 g/L with supplementation)	cofactor role in nucleic acid metabolism	[96]
Fe^2+^	adding 1 g/L FeSO_4_ increased cordycepin by ~70% (to ~596.6 mg/L)	redirects purine metabolism toward adenosine branch	[96]
Mg^2+^	present in medium (MgSO_4_·7H_2_O), supports biomass and metabolite biosynthesis	essential for enzymatic activity	[65]
Ca^2+^, K^+^, Na^+^, Se	listed as nutritional minerals present in *C. militaris* fruiting bodies and mycelia	recognized as essential nutrients	[99]

**Table 3:** Liquid-state fermentation of *Cordyceps militaris*.

Fermentation Type	C/N Ratio (Carbon: Nitrogen)	Light Wavelength	Observations	Outcome Summary	Reference
Submerged fermentation	2.66:1 (42 g/L glucose: 15.8 g/L peptone)	-	achieved maximum cordycepin production (345.4 mg/L; ~19.2 mg/L per day)	Optimal for cordycepin production with glucose as a carbon source; opimal for cell growth with galactose media	[101]
Submerged fermentation	1:1.5 (by mass)	-	adenosine & cordycepin content increased at this ratio; declined at 1:3	Reduced biomass and productivity	[18]
Submerged fermentation	8:1	-	3.5-fold increase in cordycepin production	Optimal for cordycepin production on biomass with glucose as a carbon source	[105]
Submerged fermentation	12:1	-	Maximal mycelial growth	Optimal for *C. militaris* growth	[105]
surface fermentation	-	red light (620–630 nm)	Stimulated biomass formation	Optimal for mycelial growth and adenosine accumulation	[40]
surface fermentation	-	blue light (440–450 nm)	optimal for cordycepin synthesis	Activation of purine metabolism pathways and cordycepin increase	[40]
surface fermentation	-	-	Hypoxic growth conditions	Hypoxia induced upregulation of purine metabolism	[106]

“-“ denotes missing data. Substrate concentrations are expressed as grams per 100 mL or per flask, depending on the culture method.

## Data Availability

No new data were created or analyzed in this study. Data sharing is not applicable to this article.

## Supplementary Materials

The following supporting information can be downloaded at: https://www.mdpi.com/article/10.3390/foods14193408/s1, Table S1: Integrated Summary of Bioactive Compounds, Optimal Culture Parameters, and Applications in *Cordyceps militaris*; Table S2: Comparison of Solid-State Fermentation (SSF) and liquid state Fermentation for the Production of Bioactive Compounds in *Cordyceps militaris*.
