# Supplementary material for: Influence of Culture Conditions on Bioactive Compounds in Cordyceps militaris: A Comprehensive Review
Source: Foods. 2025 Oct 1;14(19):3408. doi: 10.3390/foods14193408 (PMC12523487; doi:10.3390/foods14193408)
Supplement: Supplementary file 1 [file foods-14-03408-s001.zip › foods-3868732-supplementary.pdf]

Supplementary Table S1. Integrated Summary of Bioactive Compounds, Optimal Culture Parameters, and Applications in *Cordyceps militaris*

Compound	Optimal Cultivation Conditions	Major Bioactivities	Industrial Implications
**Cordycepin**	Moderately low C/N ratio; organic N (peptone, yeast extract); blue light; adequate DO	Anti-cancer, anti-inflammatory, immunomodulatory	High-value nutraceuticals (extracts, powders); pharmaceutical APIs (controlled SmF for reproducibility)
**Adenosine**	Red light; acidic pH (4.5–5.0); Se supplementation; organic N (yeast extract)	Anti-inflammatory, immunoregulatory via purinergic receptors	Functional foods targeting inflammation; pharma-scale adenosine enrichment
**Polysaccharides (EPS/IPS)**	Balanced C/N; hypoxia favors EPS; glucose/sucrose; trace metals	Immunostimulant (↑ cytokines, macrophages, NK cells); antioxidant; prebiotic	Functional foods, gut health supplements; pharmaceutical adjuvants
**D-Mannitol**	Mildly acidic pH; high C with limited N; stress-responsive	Antioxidant, hepatoprotective, hypolipidemic, antitumor (synergistic with chemo)	Natural antioxidant in food formulations; supportive therapy in pharmaceuticals
**Carotenoids**	Blue/pink light; oat-based medium; optimal pH/temp	Antioxidant, neuroprotective, anti-aging	Natural food colorants, nutraceuticals, cosmeceuticals
**Ergosterol**	Oxygen-dependent steps; mevalonate pathway; influenced by C/N	Anti-inflammatory, antioxidant, immunomodulatory; precursor of vitamin	Nutraceuticals; vitamin D2 fortification; biomarker for

		D2	fungal growth
**Proteins (e.g., Cordymin, CMP)**	Organic N enhances yield; blue light ↑ enzyme expression; insect substrates ↑ stress proteins	Antifungal, immunoregulatory, anticancer; extracellular enzymes (proteases, chitinases)	Functional proteins; enzyme production; pharmaceutical peptides

Supplementary Table S2. Comparison of Solid-State Fermentation (SSF) and liquid state Fermentation for the Production of Bioactive Compounds in *Cordyceps militaris*

**Parameter**	**Solid-State Fermentation (SSF)**	**Liquid state Fermentation**	**Reference**
**Cordycepin yield**	**Up to 5.62 mg/g using substrates like brown rice and millet**	**Variable; modulated by carbon and nitrogen sources; typically lower than SSF**	**[65]**
**Polysaccharide production**	**Often higher due to structural development during fruiting body formation**	**Depends on media viscosity and agitation; yields can be enhanced with optimization**	**[108]**
**Fruiting body formation**	**Efficient; necessary for certain bioactives such as cordycepin**	**Rare; primarily used for mycelial biomass rather than full fruiting body**	**[65]**

	in natural-like matrix		
Mycelial biomass	Moderate growth; less efficient for rapid biomass accumulation	High; favored for scalable production of mycelia-based extracts	[41]
Contamination risk	Higher due to exposure and complex surface environment	Lower; closed systems allow better sterility control	[20]
Process control	Difficult to regulate moisture, oxygen, and temperature uniformly	Easier to control environmental parameters (pH, nutrients, agitation, aeration)	[100]
Industrial scalability	Labor-intensive and space-consuming; scale-up is complex	High-throughput, cost-effective bioreactor systems available for large-scale production	[20,100]
Target product preference	Suitable for fruiting bodies, broader	uitable for focusing on the production of specific	[22, 44,106])

	**bioactive metabolite spectrum**		**bioactive compounds such as cordycepin, proteins, and intracellular polysaccharides, as well as for conducting metabolomics studies**	
